# Iron Overload and HFE Mutations: Are They Relevant in Cryptogenic Cirrhosis?

**DOI:** 10.5812/hepatmon.823

**Published:** 2012-02-29

**Authors:** Agustin Castiella

**Affiliations:** 1 Gastroenterology Service, Mendaro Hospital, Mendaro, Spain

**Keywords:** Iron overlood, Genetic Diseases, Genes

Dear Editor,

Hereditary hemochromatosis (HH) is the most frequent genetic disease in populations of European origin. The HH gene was cloned by Feder et al. in 1996, and 2 major mutations were discovered: C282Y and H63D. Geographical differences with mutation frequencies have been published [1][2] with a decreasing gradient of occurrence in Europe from north to south. HH leads to liver iron overload and raised liver iron concentration (LIC) is associated with liver fibrosis. When the LIC reaches 60µmol/g, or approximately twice the upper limit of the normal range (36µmol/g), activation of stellate cells appears. These cells are principally responsible for the beginning of liver fibrosis and fibrosis [3]. This process can lead to hepatic cirrhosis at the end stage. So it is of interest to ask ourselves, for those patients with cirrhosis but without any known culprit as the cause, whether iron surcharge and mutations of the HFE gene that may induce this iron overload could play a relevant role in the etiopathogenesis of cryptogenic cirrhosis.

Jowkar et al. [4] have recently published a prospective study to investigate the probable association of HFE gene mutations in a group of 100 patients with cryptogenic cirrhosis and compared the results with a control group of 50 normal, unrelated healthy individuals. No homozygotes for C282Y and H63D mutations were found, and only 22% of the patients and 28% of controls (P > 0.05) were heterozygous for the H63D mutation. Most of the cirrhotic patients and controls with this mutation did not have liver iron overload. Few studies have investigated the frequency of HFE gene mutations in patients with chronic liver diseases and cirrhosis. The results from the Jowkar et al. [4] study are similar to those published from Indian and Turkish patients [5][6]. More recently, Sikorska et al. [7] studied iron overload and HFE gene mutations in Polish patients with liver cirrhosis (61 patients), and they were compared with a control group of 42 consecutive patients subjected to liver biopsy because of chronic liver disease. They concluded that iron disorders are frequently detected in patients with liver cirrhosis, but without significant association with HFE gene mutations. Another recent study from India [8] tried to find the association of common HFE mutations and primary iron overload in liver cirrhosis. Of the 496 patients (242 cryptogenic cirrhosis) included in the study, only 13 had iron overload and only two were H63D heterozygous. The overall frequency of the H63D allele in patients and controls was not significantly different (P < 0.17).

Jowkar et al. [4], with their interesting study developed in Iran, seem to reinforce the theory that cryptogenic cirrhosis is not associated with iron overload, nor with major HFE mutations. It also appears that there are no geographical differences, since their results are similar to those published in Poland, Europe.
